# Prediction of early developmental status using neonatal structural MRI radiomics

**DOI:** 10.3389/fpubh.2026.1943195

**Published:** 2026-10-21

**Authors:** Cuimin Su, Minyu Qiu, Ying Lin, Tianyi Zhou, Xinyu Jiang, Ruming Ye, Huiyue Zhang, Yuelong Ji, Kai Yan, Qin Li, Xianghui Huang

**Affiliations:** 1 Department of Scientific Research and Education, Jinjiang Municipal Hospital, Jinjiang, China; 2 Department of Maternal and Child Health, School of Public Health, Peking University, Beijing, China; 3 School of Public Health, Shandong Second Medical University, Weifang, Shandong, China; 4 Fujian Key Laboratory of Neonatal Diseases, Xiamen Children's Hospital (Children's Hospital of Fudan University at Xiamen), Xiamen, China; 5 Xiamen Key Laboratory of Neonatal Diseases, Xiamen Children's Hospital (Children's Hospital of Fudan University at Xiamen), Xiamen, China; 6 Children's Hospital of Fudan University, National Children's Medical Center, Shanghai, China

**Keywords:** Griffiths Development Scales-Chinese (GDS-C), machine learning, neonatal structural MRI, neurodevelopmental outcome, radiomics

## Abstract

**Background:**

Early identification of developmental vulnerability in high-risk neonates remains challenging because standardized developmental assessments, such as the Griffiths Development Scales-Chinese (GDS-C), are typically performed months after birth and cannot provide objective information during the neonatal period. Structural MRI provides quantitative information regarding neonatal brain development, and radiomics may capture imaging patterns beyond conventional visual assessment. This study aimed to investigate whether neonatal structural MRI radiomics combined with clinical variables could provide early estimates of developmental status during infancy.

**Methods:**

This prospective cohort study included 93 neonates recruited from Xiamen Children's Hospital between November 2022 and August 2025. All participants underwent non-sedated brain MRI on a 3.0-T scanner during the neonatal period, and radiomic features were extracted from structural MRI. MRI radiomic features were integrated with prespecified clinical variables to develop machine learning models for estimating the Griffiths Development Scales-Chinese (GDS-C) general quotient (GQ) obtained during follow-up. Model development and performance evaluation were performed using a nested cross-validation framework involving all participants. A two-level stacking framework integrating predictions from ten regression algorithms was developed, and predictive performance was evaluated using the coefficient of determination (R^2^), root mean square error (RMSE), and mean absolute error (MAE).

**Results:**

Of the 93 neonates, 36 (38.7%) were female and 62 (66.7%) were preterm. The mean (SD) corrected age at GDS-C assessment was 7.13 (5.11) months, with a mean (SD) general quotient of 104.11 (19.77). The clinical-radiomics stacking model demonstrated moderate predictive performance under nested cross-validation, achieving an RMSE of 17.12, an MAE of 13.55, and an R^2^ of 0.24. Compared with the clinical-only stacking model, incorporation of MRI radiomic features improved prediction performance. Exploratory analyses of GDS-C subdomains and hierarchical stacking approaches were additionally performed and should be interpreted as preliminary findings.

**Conclusions:**

Neonatal MRI radiomics combined with clinical variables may provide complementary information for estimating developmental status during infancy among high-risk neonates undergoing clinically indicated MRI. These findings support the potential of quantitative imaging approaches as adjunctive tools for early developmental surveillance rather than replacements for standardized assessments. Larger multicenter studies with longitudinal follow-up are required to further evaluate clinical utility.

## 1 Introduction

The global trend toward delayed childbearing has contributed to a rising prevalence of high-risk births, including preterm delivery and low birth weight, imposing a substantial long-term burden of neurodevelopmental disease (1–3). The first year of life represents the most dynamic phase of human brain development, characterized by rapid structural maturation and unparalleled neuroplasticity (4). However, this rapid developmental trajectory renders the immature brain particularly susceptible to prenatal and perinatal insults (5, 6). This vulnerability is especially pronounced in neonates requiring hospitalization due to various medical complications, placing them at significantly increased risk of cognitive, motor, or behavioral difficulties later in childhood (7). Because clinical assessments in early infancy are often subjective and lack predictive stability (8), there is a critical need for objective biomarkers capable of identifying neurodevelopmental risks during this early window to guide timely intervention.

Currently, standardized tools such as the Bayley Scales of Infant and Toddler Development and the Gesell Developmental Schedules serve as the primary methods for evaluating neurodevelopment (9–11). In China, the Griffiths Development Scales-Chinese (GDS-C) is particularly favored for its comprehensive assessment across multiple functional domains. Although the GDS-C provides valuable information regarding developmental performance, it is typically performed months after birth and therefore cannot provide immediate insight during the neonatal period, when early identification of developmental vulnerability may be clinically important (12). Furthermore, developmental scores obtained during early infancy reflect dynamic developmental processes and may require longitudinal follow-up for a more comprehensive understanding of individual developmental trajectories (13, 14). Therefore, objective biomarkers available during the neonatal period may complement standardized developmental assessments by providing earlier information regarding subsequent developmental variation.

In the search for such objective biomarkers, magnetic resonance imaging (MRI) has emerged as an essential tool due to its non-invasive nature and high soft-tissue contrast. Specifically, T1-weighted structural MRI (sMRI), provides detailed information on brain morphology, tissue intensity patterns, and regional maturation, enabling the detection of subtle developmental alterations that may precede overt clinical symptoms (15, 16). However, conventional visual assessment of MRI remains largely qualitative and may fail to capture subtle quantitative variations in brain structure. Radiomics addresses this limitation by transforming medical images into high-dimensional, mineable data, allowing for the quantification of tissue heterogeneity and structural organization beyond human visual perception (17). While emerging studies have applied radiomics-based approaches to characterize brain injury, most have focused on contemporaneous classification (e.g., diagnosing hypoxic-ischemic encephalopathy or prematurity) (10, 18, 19). The potential of early life sMRI radiomics to predict subsequent neurodevelopmental outcomes remains largely underexplored, representing a significant opportunity to bridge the gap between early imaging and later developmental assessment.

Therefore, this study aims to develop and evaluate a machine learning model integrating neonatal structural MRI radiomics with clinical variables to estimate early developmental status measured by GDS-C scores in clinically selected high-risk neonates. By establishing this objective predictive framework, we sought to provide early quantitative information regarding developmental variation before standardized developmental assessments are available, thereby supporting future individualized follow-up strategies during infancy.

## 2 Methods

### 2.1 Participants

We used data from pairs of pregnant women and their offspring enrolled in the Xiamen Children's Brain Development Cohort, an ongoing prospective cohort study conducted in Xiamen, China. Detailed descriptions of the cohort can be found elsewhere (20). In the present study, we collected maternal and neonatal data via baseline questionnaires and medical records, and GDS-C scores during routine follow-up (20). The inclusion criteria were as follows: (1) availability of MRI radiomics data and GDS-C assessments; (2) complete clinical information for both mothers and neonates; and (3) no major organic brain lesions (e.g., intracranial hemorrhage or brain tumors) on routine clinical MRI review. The exclusion criteria included: (1) contraindications to MRI; (2) inadequate MRI image quality that precluded reliable radiomics feature extraction; and (3) presence of a major medical condition affecting growth, or development. After applying the predefined inclusion and exclusion criteria, including availability of complete MRI radiomics data, GDS-C assessment, and clinical information, a total of 93 neonates (57 males and 36 females) were included in the final analysis.

The study was approved by the Clinical Ethics Committee of Xiamen Children's Hospital (Approval No. 2022026). Pregnant women provided written informed consent for themselves and their infants. This study was reported according to the TRIPOD+AI statement for prediction model studies, with the CLEAR checklist applied for radiomics-specific reporting considerations. STROBE was used for reporting the observational cohort components. Completed STROBE, TRIPOD+AI, and CLEAR checklists are provided as separate supplementary files.

### 2.2 MRI scan and radiomics feature extraction

Neonates underwent non-sedated brain MRI scans as part of routine clinical care based on physician-determined indications, including prematurity, neonatal hypoxia, jaundice, and other conditions requiring neurological evaluation. Postmenstrual age (PMA) at MRI acquisition was calculated as gestational age at birth plus chronological age at the time of MRI examination and was recorded for all participants. MRI acquisition was not performed as a research screening procedure. All MRI scans underwent visual quality assessment before radiomics extraction. No participants were excluded due to insufficient image quality or incomplete MRI acquisition. The scans were acquired on a 3T scanner (uMR 890, United Imaging) with a 64-channel head coil, with a total duration of approximately 40 min. We acquired high-resolution T1w/T2w images (0.8 mm isotropic) using AI-assisted compressed sensing (ACS) (21), which reduced acquisition time by 37.8% compared to standard BCP protocols (20, 22, 23).

Radiomics analysis was performed using whole-brain structural MRI images. The radiomics region of interest (ROI) was defined as the entire brain parenchyma rather than atlas-based anatomical regions or manually delineated structures. An Otsu-based preliminary mask was generated to support N4 bias-field correction of each T1-weighted image using SimpleITK. The maximum number of N4 iterations was set to 20 at each of three multiresolution levels. After bias correction, a binary whole-brain mask was automatically generated using nilearn.masking.compute_brain_mask. No additional linear or nonlinear intersubject registration was performed, and radiomic features were extracted in each participant's native image space to preserve individual anatomical characteristics.

Radiomics feature extraction was performed using the PyRadiomics (version 3.1.1) (24). Before feature calculation, images and masks were resampled to isotropic 1 × 1 × 1 mm^3^ voxels. Image intensities were normalized using the built-in z-score normalization option (normalize=True). Gray-level discretization was performed using a fixed bin width of 25, and voxelArrayShift was set to 1,000. Shape features were calculated from the whole-brain mask. First-order and texture features, including gray-level co-occurrence matrix, gray-level dependence matrix, gray-level run-length matrix, gray-level size-zone matrix, and neighboring gray-tone difference matrix features, were extracted from original images and wavelet- and Laplacian-of-Gaussian-filtered images. For Laplacian-of-Gaussian filtering, sigma values of 1, 3, and 5 mm were applied. A total of 1,064 radiomic features were generated for subsequent modeling. Because the ROI was automatically generated without manual segmentation, inter-rater reliability assessment using intraclass correlation coefficients was not applicable.

### 2.3 Outcome measures

Trained physicians administered the GDS-C assessments in accordance with the standardized cohort protocol (20). For each participant, the earliest completed GDS-C score was used as the neurodevelopmental outcome. No blinding procedures were used during developmental outcome assessment. Scores were interpreted according to the age-specific normative standards provided for the corresponding assessment age. For preterm infants, developmental assessment was performed based on corrected age, whereas chronological age was used for term infants. All GDS-C domain scores and the total developmental quotient were derived and interpreted according to the corresponding age-specific standards to account for differences in maturational stage between preterm and term infants. We employed the 0–2 year version, which includes 5 domains: Movement [A] (assessing a child's gross motor skills, balance, and coordination), Individual-Society [B] (assessing a child's self-care ability and interactions with peers and adults), Language [C] (assessing a child's receptive and expressive language skills), Hand-eye Coordination [D] (assessing fine motor skills, finger dexterity, and visual tracking), and Performance [E] (assessing a child's visual-spatial ability, including processing speed and accuracy) (25). Raw domain scores were converted to developmental age equivalents and percentiles. Domain-specific developmental quotients (DQs) were calculated as developmental age divided by chronological age, multiplied by 100. For preterm infants, corrected age was used for developmental quotient calculation according to the GDS-C scoring standard. Corrected age at GDS-C assessment was recorded for all participants. The general quotient (GQ) was calculated as the mean of the 5 domain DQs (25). According to the GDS-C, when GQ ≥85, it indicates normal development (25). Children with a GQ <70, indicating significant delays in two or more developmental areas, were diagnosed with Global Developmental Delay (GDD) (25).

### 2.4 Other data collection

Neonatal (sex, birth weight, and postmenstrual age) and maternal/perinatal characteristics (maternal age, ethnicity, pregnancy-related conditions and mode of delivery) were obtained from baseline pregnancy questionnaires and electronic medical records. Completeness of predefined clinical variables was verified before statistical analysis. All clinical variables were available for the included participants, and no missing-data imputation was required.

### 2.5 Prediction model development

The primary objective of this study was to develop a machine learning model integrating neonatal brain MRI radiomic features and clinical variables to estimate subsequent developmental performance measured by the GDS-C general quotient (GQ). Considering that different GDS-C subdomains may represent distinct aspects of early neurodevelopment, the same prediction framework was additionally applied to the five GDS-C subscale scores (A–E), with comparisons between clinical-only and clinical-radiomics models performed as secondary exploratory analyses.

Given the limited sample size, all model development and performance evaluation were conducted using a nested cross-validation framework involving all participants. A 5-fold outer cross-validation loop was used for unbiased performance estimation, while a 5-fold inner cross-validation loop was applied for model training, feature selection, and hyperparameter optimization. The outer validation folds were completely isolated from all model development procedures. Within each outer training fold, preprocessing, feature selection, model fitting, and generation of stacking meta-features were performed exclusively using the available training data, whereas the corresponding outer validation fold was used only for final performance evaluation.

#### 2.5.1 Primary model development: clinical-radiomics stacking model

For the primary prediction model, radiomic features extracted from neonatal structural MRI were combined with prespecified clinical variables. A two-level stacking framework was developed. At the first level, ten regression algorithms were trained in parallel, including random forest (RF), extreme gradient boosting (XGBoost), gradient boosting machine (GBM), support vector regression (SVR), k-nearest neighbors (kNN), partial least squares regression (PLS), neural networks, elastic net, least absolute shrinkage and selection operator (LASSO), and ridge regression. The predictions generated by these base learners were subsequently integrated using a GBM meta-learner at the second level to generate the final prediction.

Radiomic feature preprocessing and selection were performed exclusively within the inner cross-validation loop. Specifically, training-data-driven Winsorization, near-zero variance filtering, correlation-based filtering, and recursive feature elimination with cross-validation (RFECV) were sequentially applied to identify informative radiomic features while minimizing information leakage. Prespecified clinical variables were retained without automated selection and included four domains: (1) sociodemographic characteristics, (2) maternal metabolic and inflammatory factors, (3) fetal growth parameters, and (4) neonatal complications.

To avoid leakage during stacking, out-of-fold predictions from the first-level models were generated within the inner cross-validation loop and used as meta-features for training the GBM meta-learner. Predictions for outer validation folds were obtained only after refitting the first-level models using the complete outer training data. The same stacking procedure was applied independently for each prediction target.

#### 2.5.2 Comparator model: clinical-only model

To evaluate the incremental predictive value of MRI radiomic features, a clinical-only stacking model was developed using the same modeling framework. This comparator model included only the prespecified clinical variables, while radiomic features were excluded. All preprocessing, model training, stacking procedures, and performance evaluations were conducted using the identical nested cross-validation framework.

#### 2.5.3 Secondary domain prediction

To investigate whether neonatal MRI radiomics could predict specific developmental domains, the same nested cross-validation stacking framework was independently applied to the five GDS-C subscale scores: Movement (A), Individual-Society (B), Language (C), Hand-eye Coordination (D), and Performance (E). These analyses were considered exploratory and were not used to define the primary prediction model.

#### 2.5.4 Exploratory hierarchical stacking analysis

As a supplementary sensitivity analysis, a hierarchical stacking strategy was additionally evaluated using a fixed training-test split approach. In this framework, the five GDS-C domain scores were first predicted separately. For each domain, predictions generated by the ten first-level algorithms were combined as domain-specific meta-features. These features were subsequently concatenated and used as inputs for a second-level model to predict the GDS-C general quotient (GQ). Performance of this hierarchical stacking approach was evaluated in the held-out test cohort using RMSE, MAE, and R^2^.

### 2.6 Statistical analysis

All statistical analyses were performed using R version 4.3.3. A fixed random seed was set before cross-validation partitioning and model training to ensure reproducibility of data splitting and computational procedures.

Predictive performance was evaluated based on the out-of-fold predictions generated from the outer cross-validation procedure. The primary evaluation metrics included root mean square error (RMSE), mean absolute error (MAE), and coefficient of determination (R^2^). Lower RMSE and MAE values indicate smaller prediction errors, whereas higher R^2^ values indicate better agreement between predicted and observed developmental scores.

For each prediction target, including the GDS-C general quotient (GQ) and the five GDS-C subscale scores (A-E), stacked models were evaluated using two input strategies: (1) clinical variables only and (2) clinical variables combined with MRI radiomic features. The incremental predictive value of MRI radiomic features was assessed by comparing the clinical-radiomics model with the clinical-only model using RMSE, MAE, and R^2^ derived from outer-fold predictions. The results for GQ were considered the primary analysis, whereas comparisons for individual GDS-C subscale scores were considered exploratory secondary analyses.

The exploratory hierarchical stacking analysis for GQ prediction was evaluated separately using the predefined held-out test cohort. Performance was assessed using RMSE, MAE, and R^2^.

## 3 Results

A total of 93 neonates (57 males and 36 females) were included in the analysis. The mean (SD) gestational age was 34.9 (3.6) weeks, with 66.7% born preterm, and the mean birth weight was 2,300.37 (859.19) g. Cesarean delivery occurred in 75.3% of cases, and 19.4% were conceived via *in vitro* fertilization. Common neonatal comorbidities included jaundice (60.2%), respiratory distress syndrome (32.3%), and retinopathy of prematurity (11.8%). Regarding maternal characteristics, the mean (SD) age at delivery was 31.1 (5.2) years; prevalent complications included inflammation (64.5%), gestational hypertension (17.2%), and gestational diabetes mellitus (23.7%) (Table 1).

**Table 1:** Basic characteristics of the study subjects.

Characteristics	Overall (*N* = 93)
Infant and maternal demographics	
Female, No. (%)	36 (38.7)
Gestational age, wk	34.85 (3.64)
Preterm birth, No. (%)	62 (66.7)
Birth weight, g	2,300.37 (859.19)
Cesarean section, No. (%)	70 (75.3)
*In vitro* fertilization, No. (%)	18 (19.4)
Neonatal jaundice, No. (%)	56 (60.2)
Neonatal respiratory distress syndrome, No. (%)	30 (32.3)
Retinopathy of prematurity, No. (%)	11 (11.8)
Maternal age, y	31.05 (5.15)
Maternal inflammation, No. (%)	60 (64.5)
Gestational hypertension, No. (%)	16 (17.2)
Gestational diabetes mellitus, No. (%)	22 (23.7)
Age at assessment	
PMA at MRI, wk	38.58 (2.68)
GDS–C assessment, mo	7.13 (5.11)
Neurodevelopmental assessment (GDS–C)	
General quotient(GQ)	104.11 (19.77)
Movement [A] (DQ)	91.68 (25.73)
Individual–Society [B] (DQ)	108.09 (25.70)
Language [C] (DQ)	130.43 (34.33)
Hand–eye Coordination [D] (DQ)	99.89 (26.52)
Performance [E] (DQ)	90.47 (25.47)

Values are mean (SD); categorical variables are shown as no. (%).

GDS–C, Griffiths Development Scales–Chinese; PMA, postmenstrual age.

The MRI examinations were performed during the neonatal period, with a mean PMA at scan of 38.58 weeks (SD, 2.68), a median PMA of 37.90 weeks (IQR, 36.90–40.00 weeks). The GDS-C assessment was performed according to corrected age in preterm infants. The median corrected age at assessment was 5.0 months (interquartile range [IQR], 3.9–8.2 months; mean ± SD, 7.13 ± 5.11 months). Most infants underwent assessment during early infancy, with 48 (51.6%) assessments performed before 6 months of corrected age, 34 (36.6%) between 6 and 12 months, and 11 (11.8%) after 12 months. The mean (SD) general quotient was 104.11 (19.77). Mean (SD) subscale developmental quotients were 91.68 (25.73) for Movement 108.09 (25.70) for Individual-Society, 130.43 (34.33) for Language, 99.89 (26.52) for Hand-eye Coordination, and 90.47 (25.47) for Performance (Table 1).

Using the nested cross-validation framework, the primary GDS-C general quotient (GQ) prediction models were evaluated based on out-of-fold predictions generated from the outer validation loops. The predictive performance of models using clinical variables alone and models integrating clinical variables with MRI radiomic features is summarized in Table 2 and Figure 1.

**Table 2:** Performance of clinical–only and clinical–radiomics stacked models for prediction of the GDS–C general quotient using nested cross–validation.

Model	Algorithm	All Set (*n* = 93)		
		RMSE	MAE	R^2^
Clinical–only	XGBoost	19.29	15.61	0.04
Clinical+Radiomics		17.37	13.94	0.22
Clinical–only	SVR	19.43	15.21	0.02
Clinical+Radiomics		19.20	14.86	0.05
Clinical–only	Ridge	19.42	15.68	0.02
Clinical+Radiomics		18.89	14.92	0.08
Clinical–only	Lasso	20.12	16.08	−0.05
Clinical+Radiomics		19.59	15.54	0.01
Clinical–only	Elastic net	20.17	16.18	−0.05
Clinical+Radiomics		19.38	15.31	0.03
Clinical–only	RF	17.71	14.37	0.19
Clinical+Radiomics		16.74	12.96	0.27
Clinical–only	PLS	20.07	16.18	−0.04
Clinical+Radiomics		19.53	15.56	0.01
Clinical–only	Neural networks	19.87	15.98	−0.02
Clinical+Radiomics		19.89	15.96	−0.02
Clinical–only	KNN	20.28	16.18	−0.06
Clinical+Radiomics		19.74	15.70	−0.01
Clinical–only	GBM	19.11	15.70	0.06
Clinical+Radiomics		16.29	13.08	0.31
Clinical–only	Stacking	19.71	15.94	−0.01
Clinical+Radiomics		17.12	13.55	0.24

**Figure 1:**
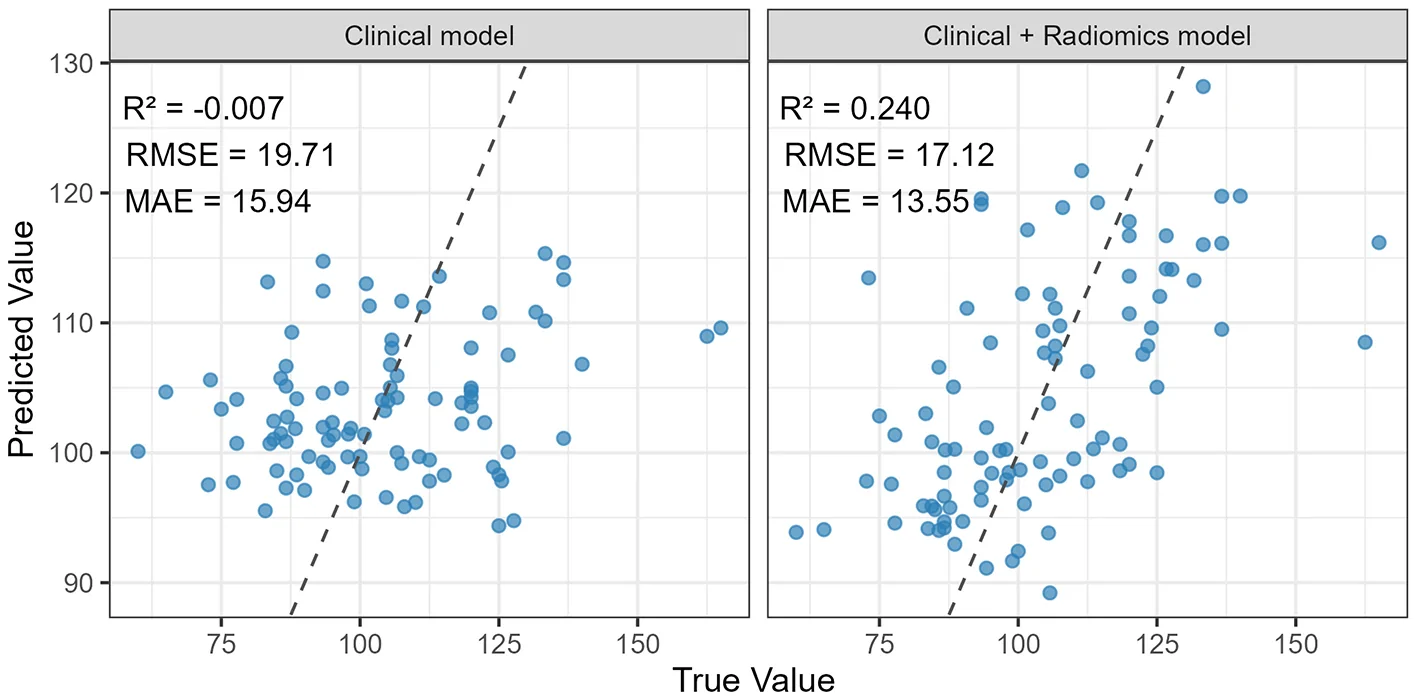
Predictive performance of clinical-only and clinical-radiomics stacking models for the GDS-C general quotient. Scatter plots show observed versus out-of-fold predicted GDS-C general quotient (GQ) scores generated from nested cross-validation. Each point represents one participant, and the dashed line indicates perfect agreement (y = x). The left and right panels show predictions from the clinical-only stacking model and the clinical+radiomics stacking model, respectively. Model performance was summarized using RMSE, MAE, and R^2^. Abbreviations: GQ, general quotient; RMSE, root mean square error; MAE, mean absolute error; R^2^, coefficient of determination.

The clinical-radiomics stacking model demonstrated improved predictive performance compared with the clinical-only stacking model for prediction of the GDS-C general quotient (GQ) (Table 2, Figure 1). Incorporation of MRI radiomic features reduced the RMSE from 19.71 to 17.12 and increased the R^2^ from−0.01 to 0.24. The final clinical-radiomics stacking model achieved an RMSE of 17.12, MAE of 13.55, and R^2^ of 0.24, indicating that MRI radiomic features provided additional predictive information beyond clinical variables alone.

As an exploratory analysis of model architecture, ten first-level algorithms and the stacked framework were compared within the same nested cross-validation procedure. As shown in Figure 2, individual algorithms demonstrated heterogeneous predictive performance, with variation in RMSE across modeling approaches. The stacked model showed competitive prediction performance compared with individual base algorithms in the evaluated cross-validation framework. However, the magnitude of performance differences between algorithms was variable, and the observed improvement should be interpreted in the context of the limited sample size and the nested cross-validation estimates.

**Figure 2:**
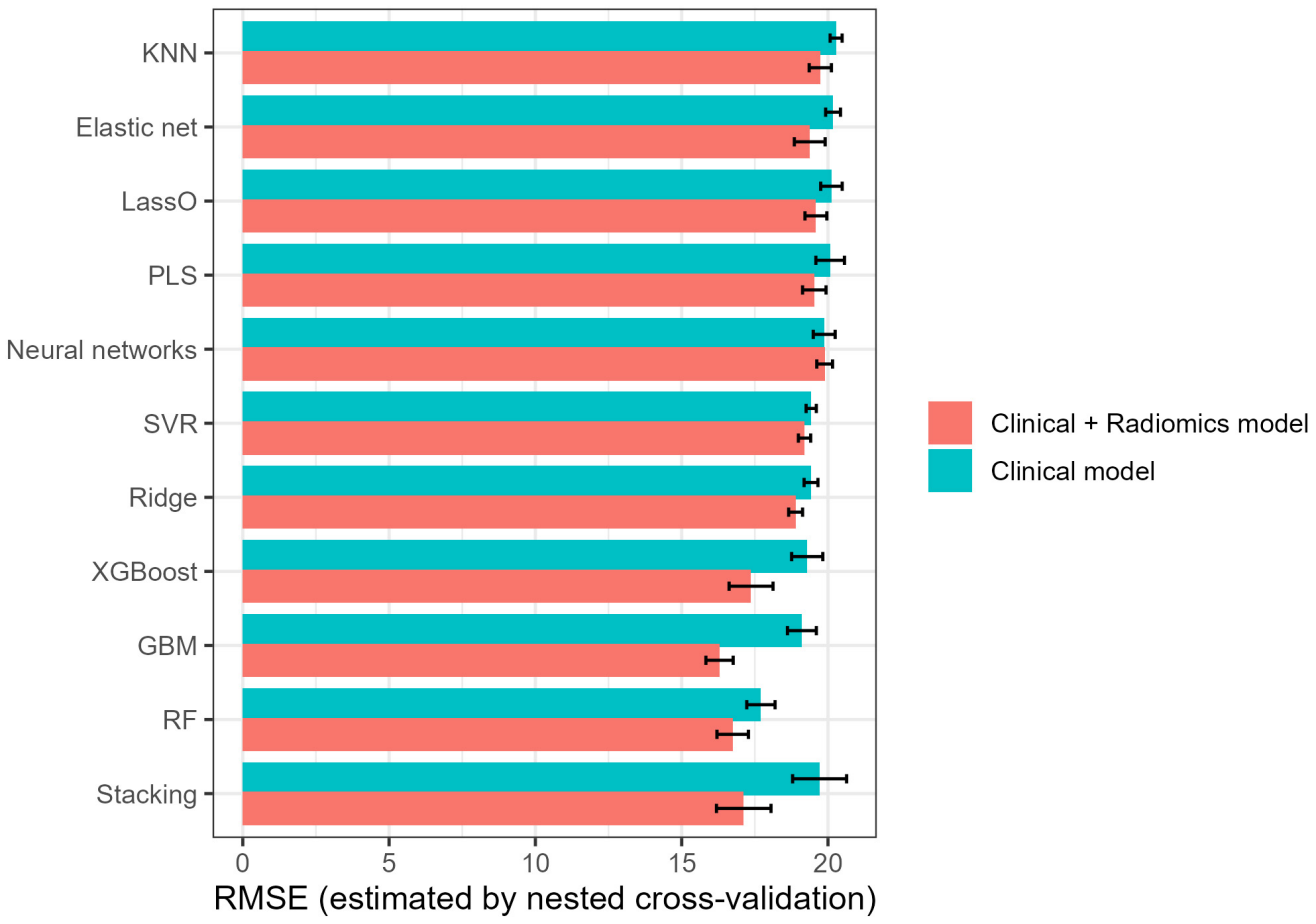
RMSE estimates obtained from nested cross-validation for individual algorithms and the stacked framework. The x-axis represents the root mean squared error (RMSE) calculated from the outer-loop validation predictions of the nested cross-validation procedure, and the y-axis indicates the evaluated modeling approaches. Lower RMSE values indicate smaller prediction errors.

For the five GDS-C subscale scores, clinical-radiomics and clinical-only stacking models were compared using the same nested cross-validation framework. The performance metrics for each developmental domain are summarized in Supplementary Table 1. These analyses were considered exploratory because of the limited sample size and multiple prediction targets.

In the supplementary sensitivity analysis, the performance metrics of the hierarchical stacking model in the training and held-out test cohorts are summarized in Supplementary Table 2. The model yielded an RMSE of 14.97, MAE of 12.06, and R^2^ of 0.53 in the held-out test cohort. Given that this analysis relied on a single data partition, these estimates were interpreted as exploratory and were not considered the primary measure of model generalizability.

## 4 Discussion

This study investigated whether neonatal structural MRI radiomics combined with clinical variables could provide early quantitative information regarding developmental status assessed during infancy among clinically selected high-risk neonates. Using a nested cross-validation framework, the clinical-radiomics stacking model demonstrated moderate predictive performance for the GDS-C general quotient, with improved performance compared with a clinical-variable-only model. These findings suggest that MRI-derived quantitative features may provide additional information beyond routinely collected clinical factors for estimating developmental variation during infancy. Importantly, this study does not aim to replace standardized developmental assessments, but rather explores whether information obtained shortly after birth may contribute to earlier identification of infants who warrant closer developmental surveillance.

Early identification of neurodevelopmental risk is a pivotal priority in pediatrics, particularly for neonates exposed to perinatal insults such as hypoxia that may compromise brain integrity; however, predictive models relying solely on clinical or demographic factors have historically demonstrated suboptimal performance. For example, England-Mason et al. (26). analyzed a limited number of chemical exposures, as well as sociodemographic factors and maternal mental health, developing and validating a machine learning model for predicting children's Bayley-III cognitive, language, and motor outcomes at 2 years of age, where cognitive scores were best predicted by 29 variables (R^2^ = 0.07, RMSE = 13.45), language outcomes by 45 variables (R^2^ = 0.09, RMSE = 13.83), and motor outcomes by 33 variables (R^2^ = 0.09, RMSE = 13.85). In contrast to these models restricted to conventional clinical or demographic factors, Jang et al. (27) used multimodal structural magnetic resonance imaging with local connectivity features and reported performance for predicting Bayley-III outcomes in the very-to-late preterm group. A RF model incorporating local connectivity and clinical features yielded an RMSE of 13.70 and explained 10% of the variance in motor scores. ElasticNet models yielded an RMSE of 11.21 (variance explained, 17%) for cognitive scores when incorporating local and global connectivity plus clinical characteristics and an RMSE of 12.43 (variance explained, 6%) for language scores when incorporating local connectivity, volumetric, and global connectivity features. Li Z et al. (28) applied a self-supervised learning approach to neonatal T2-weighted MRI radiomic data in very preterm infants and reported promising performance for cognitive outcome prediction. However, previous studies have primarily focused on specific populations, such as very preterm infants, or relied on specialized imaging-derived representations. Whether routinely acquired neonatal structural MRI can provide quantitative radiomic information for early developmental estimation in clinically heterogeneous high-risk neonates remains insufficiently explored. In the present study, the clinical-radiomics stacking model achieved an RMSE of 17.12, MAE of 13.55, and R^2^ of 0.24 for GDS-C general quotient prediction under nested cross-validation. Although direct comparison across studies is limited because of differences in populations, imaging modalities, outcomes, and validation strategies, these findings support the potential role of neonatal MRI-derived quantitative features in early developmental prediction.

Standardized developmental assessments such as the GDS-C provide valuable information regarding infant developmental performance, but they are generally performed months after birth. Although the Language domain showed a relatively higher mean DQ than the other domains in our cohort, GQ is derived from the average DQ across all five subdomains, assessed by the same examiner in a single session. As an integral component of the standardized GQ, the higher Language DQ may indicate relatively stronger early communicative skills in this cohort and contributes to the overall GQ as part of its developmental profile. Accordingly, GQ was interpreted as a standardized composite measure of early developmental status rather than as an indicator of any single developmental domain.

Accordingly, the present study should be considered an investigation of early developmental estimation rather than a model for predicting irreversible neurodevelopmental disorders. Future studies incorporating longitudinal developmental assessments are needed to determine whether neonatal MRI-based radiomics can predict persistent developmental outcomes over longer follow-up periods.

The high dimensionality and collinearity inherent to radiomics make dimensionality reduction a critical step for developing robust predictive models. In this study, RFECV was implemented as an embedded dimensionality reduction procedure within the nested cross-validation framework to reduce feature redundancy and control model complexity (29). Such dimensionality control is particularly important in small-to-moderate neonatal imaging cohorts, where the feature-to-sample ratio is otherwise unfavorable and model overfitting is a concern (30). While radiomic feature extraction and dimensionality reduction facilitated the development of prediction models, clinical interpretability remains an important challenge for translating radiomics-based prediction models into clinical research and practice.

Many of the incorporated features, particularly those derived from wavelet transforms or higher-order filters, lack clear and intuitive histopathological correlates (29, 31). This abstraction creates a ‘semantic gap' between quantitative imaging data and biological reality, making it challenging for clinicians to visually validate the model's decision-making logic. Future research should prioritize the refinement of radiomic features through the lens of clinical interpretability.

Beyond MRI-derived quantitative features, neurodevelopment is profoundly shaped by the broader clinical context. To capture this, we intergrated prespecified perinatal and early-life variables spanning four domains: sociodemographic, maternal metabolic and inflammatory factors, fetal growth and neonatal complications. These covariates represent complementary clinical factors associated with neonatal condition and developmental trajectories and were measured prior to outcome assessment (32–39). Integration of MRI radiomic features with clinical variables improved predictive performance compared with clinical variables alone, suggesting that quantitative imaging features may provide complementary information regarding developmental variation. The proposed framework is not intended to replace established neurological examinations or developmental surveillance tools, such as neonatal neurobehavioral assessments or general movements assessment. Instead, MRI-based prediction may serve as an adjunctive approach to identify infants who may help prioritize infants for closer follow-up when functional differences are not yet apparent. Future studies integrating imaging biomarkers with clinical assessments and longitudinal outcomes are needed to determine their incremental clinical utility.

However, several limitations should be acknowledged regarding the application of our model. First, the relatively small sample size may introduce sampling variability, limit statistical power, and increase the risk of overfitting, thereby reducing generalizability. To reduce the risk of optimistic performance estimates, we implemented nested cross-validation, in which preprocessing, feature selection, and model optimization were were performed exclusively within the inner loops, while model performance was estimated using the outer validation folds (40, 41). This strategy provided a more conservative estimate of model performance (42). Although the stacked framework demonstrated promising but preliminary performance within the nested cross-validation framework, its incremental benefit over simpler models remains uncertain, particularly in small datasets. Therefore, stacking should be considered a promising modeling strategy rather than a definitive superior approach until validated in larger independent cohorts. Future studies with larger cohorts and multicenter designs are therefore needed to fully validate model robustness, particularly across diverse clinical settings.

Second, our study cohort was predominantly composed of vulnerable neonates admitted to the NICU, characterized by a high prevalence of prematurity (66.7%) and comorbidities such as jaundice, respiratory distress syndrome, and retinopathy of prematurity. Consequently, our findings may not be generalizable to the healthy, full-term newborn population. However, brain MRI is not routinely performed as a screening procedure in low-risk infants without clinical indications and is generally reserved for neonates with specific neurological concerns or increased risk of brain injury (43). Therefore, while the model's applicability to the general population is limited, it may be more relevant to the specific demographic that clinically warrants neuroimaging—namely, high-risk neonates who face the clinically relevant uncertainty regarding their neurodevelopmental trajectory, providing a potential framework for early developmental risk stratification among clinically vulnerable neonates, although its ability to identify specific children requiring intervention remains to be established.

Third, all MRI examinations were performed at a single center using a single scanner and a standardized acquisition protocol, including an AI-assisted compressed-sensing reconstruction approach. Although this strategy ensured consistency of image acquisition and reduced within-study variability, radiomic features may be influenced by scanner hardware, acquisition parameters, reconstruction algorithms, and preprocessing pipelines. Therefore, the reproducibility and generalizability of the identified radiomic signatures across different MRI systems and institutions remain uncertain. Future multicenter studies with harmonized acquisition protocols, phantom-based calibration, or feature robustness assessment across different scanners and reconstruction methods are needed to determine the portability of MRI radiomics models in clinical practice. In addition, although MRI examinations were performed within the neonatal period according to clinical indications, the PMA at MRI acquisition was not completely uniform across participants. Given that neonatal brain structure undergoes rapid maturation during this developmental window, variation in PMA may introduce residual biological heterogeneity into radiomic features. Although the majority of scans were obtained near term-equivalent age, future studies with standardized MRI acquisition windows or larger longitudinal datasets are needed to further clarify the influence of developmental timing on neonatal radiomic signatures.

Fourth, the developmental outcome was assessed using a single GDS-C evaluation, and the timing of assessment varied according to the cohort follow-up schedule. Although GDS-C scores were calculated based on age-adjusted developmental norms and corrected age was used for preterm infants, the corrected age at assessment ranged from 2 to 27 months, with the majority of assessments performed during early infancy. Assessment age was not included as a predictor in model development because the objective of this study was to estimate subsequent developmental status using information available during the neonatal period, rather than variables obtained at the time of outcome evaluation. Nevertheless, variability in assessment timing may introduce additional heterogeneity in developmental outcome measurement and should be considered when interpreting model performance. Future studies using standardized assessment windows and longitudinal developmental follow-up are needed to further evaluate the robustness of MRI-based prediction across developmental stages. Additionally, future clinical implementation should consider appropriate communication strategies to ensure that predictive estimates are interpreted as probabilistic information rather than definitive outcomes.

## 5 Conclusions

In conclusion, this study suggests that neonatal MRI radiomics combined with clinical variables may provide additional information for estimating developmental status during infancy among high-risk neonates who undergo clinically indicated MRI. These findings highlight the potential of quantitative imaging approaches as complementary tools for early developmental surveillance rather than replacements for standardized assessments. However, given the limited sample size, single-center design, and lack of external validation, larger multicenter studies with longitudinal follow-up are required before potential clinical translation.

## Data Availability

The datasets generated and analyzed during the current study are not publicly available due to ethical and information governance restrictions. Requests to access de-identified data extracts should be directed to the Xiamen Children's Hospital Ethics Board and are subject to institutional approval. The R code used for the analyses is available from the corresponding author upon reasonable request. Requests to access the datasets should be directed to Qin Li, qin1018@pku.edu.cn.
