# Supplementary material for: Prediction of early developmental status using neonatal structural MRI radiomics
Source: Front Public Health. 2026 Oct 21;14:1943195. doi: 10.3389/fpubh.2026.1943195 (PMC13731250; doi:10.3389/fpubh.2026.1943195)
Supplement: Supplementary file 1 [file Data_Sheet_1.pdf]

STROBE Statement—Checklist of items that should be included in reports of ***cohort studies***

	Item No	Recommendation	Location
**Title and abstract**	1	(*a*) Indicate the study's design with a commonly used term in the title or the abstract	Title page and Abstract
		(*b*) Provide in the abstract an informative and balanced summary of what was done and what was found	Title page and Abstract
**Introduction**			
Background/rationale	2	Explain the scientific background and rationale for the investigation being reported	Introduction
Objectives	3	State specific objectives, including any prespecified hypotheses	final paragraph of the Introduction
**Methods**			
Study design	4	Present key elements of study design early in the paper	Methods—Participants
Setting	5	Describe the setting, locations, and relevant dates, including periods of recruitment, exposure, follow-up, and data collection	Methods—Participants
Participants	6	(*a*) Give the eligibility criteria, and the sources and methods of selection of participants. Describe methods of follow-up	Methods—Participants
		(*b*) For matched studies, give matching criteria and number of exposed and unexposed	N/A
Variables	7	Clearly define all outcomes, exposures, predictors, potential confounders, and effect modifiers. Give diagnostic criteria, if applicable	Methods—MRI Scan and Radiomics Feature Extraction, Outcome Measures and Other Data Collection
Data sources/ measurement	8*	For each variable of interest, give sources of data and details of methods of assessment (measurement). Describe comparability of assessment methods if there is more than one group	Methods—MRI Scan and Radiomics Feature Extraction, Outcome Measures, and Other Data Collection
Bias	9	Describe any efforts to address potential sources of bias	Methods—Prediction Model Development, and Statistical Analysis
Study size	10	Explain how the study size was arrived at	Methods—Participants
Quantitative variables	11	Explain how quantitative variables were handled in the analyses. If applicable, describe which groupings were chosen and why	Methods—Outcome Measures and Statistical Analysis
Statistical methods	12	(*a*) Describe all statistical methods, including those used to control for confounding	Methods—Prediction Model Development and Statistical Analysis
		(*b*) Describe any methods used to examine subgroups and interactions	N/A
		(*c*) Explain how missing data were addressed	Methods—Other Data Collection
		(*d*) If applicable, explain how loss to follow-up was addressed	N/A
		(*e*) Describe any sensitivity analyses	Methods—Statistical Analysis
**Results**			
Participants	13*	(a) Report numbers of individuals at each stage of study—eg numbers potentially eligible, examined for eligibility, confirmed eligible, included in the study, completing follow-up, and analysed	Results, first paragraph

		(b) Give reasons for non-participation at each stage	N/A
		(c) Consider use of a flow diagram	N/A
Descriptive data	14*	(a) Give characteristics of study participants (eg demographic, clinical, social) and information on exposures and potential confounders	Results, first paragraph, and Table 1
		(b) Indicate number of participants with missing data for each variable of interest	Methods—Other Data Collection
		(c) Summarise follow-up time (eg, average and total amount)	Results, second paragraph, and Table 1
Outcome data	15*	Report numbers of outcome events or summary measures over time	Results and Table 1

Main results	16	(*a*) Give unadjusted estimates and, if applicable, confounder-adjusted estimates and their precision (eg, 95% confidence interval). Make clear which confounders were adjusted for and why they were included	Results, and Table 2
		(*b*) Report category boundaries when continuous variables were categorized	N/A
		(*c*) If relevant, consider translating estimates of relative risk into absolute risk for a meaningful time period	N/A
Other analyses	17	Report other analyses done—eg analyses of subgroups and interactions, and sensitivity analyses	Results, and Supplementary Tables 1-2
**Discussion**			
Key results	18	Summarise key results with reference to study objectives	Discussion, first paragraph
Limitations	19	Discuss limitations of the study, taking into account sources of potential bias or imprecision. Discuss both direction and magnitude of any potential bias	Discussion, seventh through tenth paragraphs
Interpretation	20	Give a cautious overall interpretation of results considering objectives, limitations, multiplicity of analyses, results from similar studies, and other relevant evidence	Discussion, first through sixth paragraphs
Generalisability	21	Discuss the generalisability (external validity) of the study results	Discussion, seventh paragraph
**Other information**			
Funding	22	Give the source of funding and the role of the funders for the present study and, if applicable, for the original study on which the present article is based	Funding

*Give information separately for exposed and unexposed groups.

Note: An Explanation and Elaboration article discusses each checklist item and gives methodological background and published examples of transparent reporting. The STROBE checklist is best used in conjunction with this article (freely available on the Web sites of PLoS Medicine at http://www.plosmedicine.org/, Annals of Internal Medicine at http://www.annals.org/, and Epidemiology at http://www.epidem.com/). Information on the STROBE Initiative is available at http://www.strobe-statement.org.
