# Supplementary material for: Prediction of early developmental status using neonatal structural MRI radiomics
Source: Front Public Health. 2026 Oct 21;14:1943195. doi: 10.3389/fpubh.2026.1943195 (PMC13731250; doi:10.3389/fpubh.2026.1943195)
Supplement: Supplementary file 3 [file Data_Sheet_3.pdf]

CLEAR checklist						
**Section**	**No.**	**Item**	**Yes**	**No**	**n/a**	**Location**
**Title**						
	1	Relevant title, specifying the radiomic methodology	☑	☐	☐	Tittle
**Abstract**						
	2	Structured summary with relevant information	☑	☐	☐	Abstract
**Keywords**						
	3	Relevant keywords for radiomics	☑	☐	☐	Keywords
**Introduction**						
	4	Scientific or clinical background	☑	☐	☐	Introduction
	5	Rationale for using a radiomic approach	☑	☐	☐	Introduction
	6	Study objective(s)	☑	☐	☐	final paragraph of the Introduction
**Method**						
Study Design	7	Adherence to guidelines or checklists (e.g., CLEAR checklist)	☑	☐	☐	Methods — Participants
	8	Ethical details (e.g., approval, consent, data protection)	☑	☐	☐	Methods — Participants
	9	Sample size calculation	☐	☑	☐	
	10	Study nature (e.g., retrospective, prospective)	☑	☐	☐	Methods — Participants
	11	Eligibility criteria	☑	☐	☐	Methods — Participants
	12	Flowchart for technical pipeline	☐	☑	☐	
Data	13	Data source (e.g., private, public)	☑	☐	☐	Methods — Participants
	14	Data overlap	☑	☐	☐	Methods — Prediction Model Development
	15	Data split methodology	☑	☐	☐	Methods — Prediction Model Development
	16	Imaging protocol (i.e., image acquisition and processing)	☑	☐	☐	Methods—MRI Scan and Radiomics Feature Extraction
	17	Definition of non-radiomic predictor variables	☑	☐	☐	Methods—Other Data Collection
	18	Definition of the reference standard (i.e., outcome variable)	☑	☐	☐	Methods — Outcome Measures
Segmentation	19	Segmentation strategy	☑	☐	☐	Methods — MRI Scan and Radiomics Feature Extraction
	20	Details of operators performing segmentation	☐	☐	☑	
Pre-processing	21	Image pre-processing details	☑	☐	☐	Methods — MRI Scan and Radiomics

Insights Imaging (2023) Kocak B, Baessler B, Bakas S et al.

						Feature Extraction
	22	Resampling method and its parameters	☑	☐	☐	Methods — MRI Scan and Radiomics Feature Extraction
	23	Discretization method and its parameters	☑	☐	☐	Methods — MRI Scan and Radiomics Feature Extraction
	24	Image types (e.g., original, filtered, transformed)	☑	☐	☐	Methods — MRI Scan and Radiomics Feature Extraction
Feature extraction	25	Feature extraction method	☑	☐	☐	Methods — MRI Scan and Radiomics Feature Extraction
	26	Feature classes	☑	☐	☐	Methods — MRI Scan and Radiomics Feature Extraction
	27	Number of features	☑	☐	☐	Methods — MRI Scan and Radiomics Feature Extraction
	28	Default configuration statement for remaining parameters	☑	☐	☐	Methods — MRI Scan and Radiomics Feature Extraction
Data preparation	29	Handling of missing data	☐	☑	☐	
	30	Details of class imbalance	☐	☐	☑	
	31	Details of segmentation reliability analysis	☐	☐	☑	
	32	Feature scaling details (e.g., normalization, standardization)	☑	☐	☐	Methods—Prediction Model Development
	33	Dimension reduction details	☑	☐	☐	Methods—Prediction Model Development
Modeling	34	Algorithm details	☑	☐	☐	Methods—Prediction Model Development
	35	Training and tuning details	☑	☐	☐	Methods—Prediction Model Development
	36	Handling of confounders	☑	☐	☐	Methods — Prediction Model Development
	37	Model selection strategy	☑	☐	☐	Methods — Prediction Model

						Development and Statistical Analysis
Evaluation	38	Testing technique (e.g., internal, external)	☑	☐	☐	Methods — Prediction Model Development and Statistical Analysis
	39	Performance metrics and rationale for choosing	☑	☐	☐	Methods — Statistical Analysis
	40	Uncertainty evaluation and measures (e.g., confidence intervals)	☐	☐	☑	
	41	Statistical performance comparison (e.g., DeLong's test)	☐	☐	☑	
	42	Comparison with non-radiomic and combined methods	☑	☐	☐	Methods — Statistical Analysis
	43	Interpretability and explainability methods	☐	☑	☐	
**Results**						
	44	Baseline demographic and clinical characteristics	☑	☐	☐	Results
	45	Flowchart for eligibility criteria	☐	☑	☐	
	46	Feature statistics (e.g., reproducibility, feature selection)	☐	☑	☐	
	47	Model performance evaluation	☑	☐	☐	Results
	48	Comparison with non-radiomic and combined approaches	☑	☐	☐	Results
**Discussion**						
	49	Overview of important findings	☑	☐	☐	Discussion
	50	Previous works with differences from the current study	☑	☐	☐	Discussion
	51	Practical implications	☑	☐	☐	Discussion
	52	Strengths and limitations (e.g., bias and generalizability issues)	☑	☐	☐	Discussion
**Open Science**						
Data availability	53	Sharing images along with segmentation data [n/e]	☐	☐	☑	
	54	Sharing radiomic feature data	☐	☑	☐	
Code availability	55	Sharing pre-processing scripts or settings	☐	☑	☐	
	56	Sharing source code for modeling	☑	☐	☐	Data availability statement
Model availability	57	Sharing final model files	☐	☑	☐	
	58	Sharing a ready-to-use system [n/e]	☐	☐	☑	

Yes, details provided; **No**, details not provided; **n/e**, not essential; **n/a**, not applicable

Note: Use the checklist in conjunction with the main text for clarification of all items. Fill the "Page" column with the related page number where the information is provided.
