# Supplementary material for: Prediction of early developmental status using neonatal structural MRI radiomics
Source: Front Public Health. 2026 Oct 21;14:1943195. doi: 10.3389/fpubh.2026.1943195 (PMC13731250; doi:10.3389/fpubh.2026.1943195)
Supplement: Supplementary file 4 [file Table_1.docx]

Supplementary Material

# 1 Supplementary Tables

**Supplemental table 1.** Predictive performance of clinical-radiomics and clinical-only stacking models for GDS-C subscale scores using nested cross-validation

| GDS-C  subscale | Model | Algorithm | All Set (n=93) | | |
| --- | --- | --- | --- | --- | --- |
|  |  |  | RMSE | MAE | R^2^ |
| Movement [A] | Clinical-only | XGBoost | 26.71 | 22.25 | -0.09 |
|  | Clinical+Radiomics |  | 27.24 | 21.77 | -0.13 |
|  | Clinical-only | SVR | 2482 | 20.70 | 0.06 |
|  | Clinical+Radiomics |  | 24.65 | 20.21 | 0.07 |
|  | Clinical-only | Ridge | 24.63 | 20.92 | 0.07 |
|  | Clinical+Radiomics |  | 24.10 | 19.63 | 0.11 |
|  | Clinical-only | Lasso | 25.63 | 21.45 | -0.01 |
|  | Clinical+Radiomics |  | 25.93 | 21.37 | -0.03 |
|  | Clinical-only | Elastic net | 25.35 | 21.46 | 0.02 |
|  | Clinical+Radiomics |  | 25.38 | 20.90 | 0.02 |
|  | Clinical-only | RF | 24.12 | 19.78 | 0.11 |
|  | Clinical+Radiomics |  | 24.46 | 19.97 | 0.09 |
|  | Clinical-only | PLS | 25.73 | 21.85 | -0.01 |
|  | Clinical+Radiomics |  | 24.88 | 20.34 | 0.06 |
|  | Clinical-only | Neural networks | 25.99 | 21.55 | -0.03 |
|  | Clinical+Radiomics |  | 25.91 | 21.41 | -0.02 |
|  | Clinical-only | KNN | 25.20 | 20.30 | 0.04 |
|  | Clinical+Radiomics |  | 24.98 | 20.26 | 0.05 |
|  | Clinical-only | GBM | 25.57 | 21.26 | 0.00 |
|  | Clinical+Radiomics |  | 25.98 | 21.06 | -0.03 |
|  | Clinical-only | Stacking | 25.61 | 21.32 | 0.00 |
|  | Clinical+Radiomics |  | 25.27 | 20.60 | 0.02 |
| Individual-Society [B] | Clinical-only | XGBoost | 22.86 | 17.23 | 0.20 |
|  | Clinical+Radiomics |  | 20.09 | 16.12 | 0.38 |
|  | Clinical-only | SVR | 24.00 | 18.61 | 0.12 |
|  | Clinical+Radiomics |  | 24.31 | 19.19 | 0.10 |
|  | Clinical-only | Ridge | 24.61 | 19.47 | 0.07 |
|  | Clinical+Radiomics |  | 24.47 | 19.52 | 0.08 |
|  | Clinical-only | Lasso | 24.57 | 19.47 | 0.08 |
|  | Clinical+Radiomics |  | 24.48 | 19.58 | 0.08 |
|  | Clinical-only | Elastic net | 24.85 | 19.62 | 0.05 |
|  | Clinical+Radiomics |  | 24.61 | 19.71 | 0.07 |
|  | Clinical-only | RF | 21.67 | 16.95 | 0.28 |
|  | Clinical+Radiomics |  | 19.87 | 15.52 | 0.40 |
|  | Clinical-only | PLS | 25.27 | 20.25 | 0.02 |
|  | Clinical+Radiomics |  | 24.69 | 19.69 | 0.07 |
|  | Clinical-only | Neural networks | 25.42 | 20.52 | 0.01 |
|  | Clinical+Radiomics |  | 25.17 | 20.22 | 0.03 |
|  | Clinical-only | KNN | 25.20 | 19.82 | 0.03 |
|  | Clinical+Radiomics |  | 24.59 | 19.85 | 0.07 |
|  | Clinical-only | GBM | 21.32 | 16.73 | 0.30 |
|  | Clinical+Radiomics |  | 20.20 | 15.88 | 0.38 |
|  | Clinical-only | Stacking | 22.77 | 17.84 | 0.21 |
|  | Clinical+Radiomics |  | 20.80 | 16.89 | 0.34 |
| Language [C] | Clinical-only | XGBoost | 25.98 | 20.74 | 0.42 |
|  | Clinical+Radiomics |  | 23.96 | 19.11 | 0.51 |
|  | Clinical-only | SVR | 29.78 | 24.14 | 0.24 |
|  | Clinical+Radiomics |  | 24.81 | 19.72 | 0.47 |
|  | Clinical-only | Ridge | 28.96 | 23.45 | 0.28 |
|  | Clinical+Radiomics |  | 27.36 | 23.25 | 0.24 |
|  | Clinical-only | Lasso | 29.37 | 23.76 | 0.26 |
|  | Clinical+Radiomics |  | 27.72 | 23.48 | 0.22 |
|  | Clinical-only | Elastic net | 29.33 | 23.90 | 0.26 |
|  | Clinical+Radiomics |  | 27.89 | 23.61 | 0.21 |
|  | Clinical-only | RF | 23.98 | 18.90 | 0.51 |
|  | Clinical+Radiomics |  | 24.34 | 19.14 | 0.49 |
|  | Clinical-only | PLS | 28.94 | 23.63 | 0.28 |
|  | Clinical+Radiomics |  | 28.16 | 24.00 | 0.19 |
|  | Clinical-only | Neural networks | 32.13 | 26.53 | 0.11 |
|  | Clinical+Radiomics |  | 30.66 | 25.00 | 0.19 |
|  | Clinical-only | KNN | 31.98 | 26.73 | 0.12 |
|  | Clinical+Radiomics |  | 24.53 | 19.61 | 0.48 |
|  | Clinical-only | GBM | 25.08 | 20.07 | 0.46 |
|  | Clinical+Radiomics |  | 24.30 | 19.51 | 0.49 |
|  | Clinical-only | Stacking | 25.94 | 21.21 | 0.42 |
|  | Clinical+Radiomics |  | 25.54 | 20.62 | 0.44 |
| Hand-eye Coordination [D] | Clinical-only | XGBoost | 26.86 | 21.18 | -0.04 |
|  | Clinical+Radiomics |  | 26.33 | 20.84 | 0.00 |
|  | Clinical-only | SVR | 27.44 | 21.79 | -0.08 |
|  | Clinical+Radiomics |  | 25.16 | 19.57 | 0.09 |
|  | Clinical-only | Ridge | 27.80 | 22.03 | -0.11 |
|  | Clinical+Radiomics |  | 25.57 | 19.82 | 0.06 |
|  | Clinical-only | Lasso | 26.55 | 20.92 | -0.01 |
|  | Clinical+Radiomics |  | 26.88 | 20.49 | -0.04 |
|  | Clinical-only | Elastic net | 26.48 | 20.79 | -0.01 |
|  | Clinical+Radiomics |  | 26.12 | 20.07 | 0.02 |
|  | Clinical-only | RF | 24.26 | 19.39 | 0.15 |
|  | Clinical+Radiomics |  | 24.31 | 18.99 | 0.15 |
|  | Clinical-only | PLS | 28.09 | 22.16 | -0.13 |
|  | Clinical+Radiomics |  | 26.15 | 20.50 | 0.02 |
|  | Clinical-only | Neural networks | 26.61 | 20.94 | -0.02 |
|  | Clinical+Radiomics |  | 26.87 | 21.35 | -0.04 |
|  | Clinical-only | KNN | 28.54 | 22.48 | -0.17 |
|  | Clinical+Radiomics |  | 26.36 | 20.32 | 0.00 |
|  | Clinical-only | GBM | 27.63 | 21.92 | -0.10 |
|  | Clinical+Radiomics |  | 26.14 | 20.85 | 0.02 |
|  | Clinical-only | Stacking | 26.32 | 20.82 | 0.00 |
|  | Clinical+Radiomics |  | 26.40 | 20.60 | -0.00 |
| Performance [E] | Clinical-only | XGBoost | 26.50 | 20.53 | -0.10 |
|  | Clinical+Radiomics |  | 24.95 | 18.36 | 0.03 |
|  | Clinical-only | SVR | 25.70 | 20.02 | -0.03 |
|  | Clinical+Radiomics |  | 24.74 | 19.44 | 0.05 |
|  | Clinical-only | Ridge | 25.97 | 20.16 | -0.05 |
|  | Clinical+Radiomics |  | 24.47 | 18.97 | 0.07 |
|  | Clinical-only | Lasso | 26.11 | 20.17 | -0.06 |
|  | Clinical+Radiomics |  | 24.81 | 19.28 | 0.04 |
|  | Clinical-only | Elastic net | 26.16 | 20.32 | -0.07 |
|  | Clinical+Radiomics |  | 24.77 | 19.30 | 0.04 |
|  | Clinical-only | RF | 23.48 | 18.61 | 0.14 |
|  | Clinical+Radiomics |  | 22.95 | 16.66 | 0.18 |
|  | Clinical-only | PLS | 27.80 | 21.61 | -0.21 |
|  | Clinical+Radiomics |  | 24.62 | 19.15 | 0.06 |
|  | Clinical-only | Neural networks | 26.00 | 19.90 | -0.05 |
|  | Clinical+Radiomics |  | 25.00 | 19.22 | 0.03 |
|  | Clinical-only | KNN | 26.63 | 20.82 | -0.11 |
|  | Clinical+Radiomics |  | 25.33 | 19.20 | 0.00 |
|  | Clinical-only | GBM | 25.22 | 19.44 | 0.01 |
|  | Clinical+Radiomics |  | 24.05 | 17.72 | 0.10 |
|  | Clinical-only | Stacking | 25.97 | 20.40 | -0.05 |
|  | Clinical+Radiomics |  | 25.78 | 19.05 | -0.04 |

Performance metrics were obtained from nested cross-validation evaluation of clinical-radiomics and clinical-only stacking models for prediction of GDS-C subscale scores. These analyses were exploratory because of the limited sample size and multiple prediction targets. Abbreviations: RMSE, root mean squared error; MAE, mean absolute error; R^2^, coefficient of determination; GDS-C, Griffiths Development Scales-Chinese.

**Supplemental table 2.** Predictive performance of individual machine learning models and the hierarchical stacking framework for predicting the GDS-C general quotient in the split-sample evaluation

| Algorithm | Training Set (n=70) | | | Test Set (n=23) | | |
| --- | --- | --- | --- | --- | --- | --- |
|  | RMSE | MAE | R^2^ | RMSE | MAE | R^2^ |
| GBM | 4.02 | 3.06 | 0.96 | 14.97 | 12.06 | 0.53 |
| Neural networks | 9.74 | 6.14 | 0.77 | 16.37 | 13.98 | 0.43 |
| Elastic net | 12.99 | 10.60 | 0.54 | 20.69 | 17.47 | 0.13 |
| XGBoost | 3.63 | 2.23 | 0.97 | 17.24 | 14.70 | 0.45 |
| PLS | 15.79 | 13.21 | 0.32 | 21.52 | 18.73 | 0.09 |
| KNN | 14.44 | 11.68 | 0.45 | 22.80 | 19.62 | 0.02 |
| SVR | 16.38 | 12.20 | 0.31 | 19.51 | 15.64 | 0.20 |
| Lasso | 12.82 | 10.34 | 0.55 | 20.35 | 17.14 | 0.15 |
| Ridge | 14.40 | 11.82 | 0.44 | 20.60 | 17.83 | 0.13 |
| RF | 2.91 | 2.25 | 0.99 | 17.77 | 14.89 | 0.40 |

Predictive performance was evaluated using the original split-sample approach, with the dataset divided into training and testing sets. Performance metrics included root mean squared error (RMSE), mean absolute error (MAE), and coefficient of determination (R²). Higher R² values and lower RMSE and MAE values indicate better predictive accuracy. These results represent the initial split-sample evaluation and are provided as supplementary information, whereas the primary model performance evaluation was based on nested cross-validation. Abbreviations: SVR, support vector regression; GBM, gradient boosting machine; PLS, partial least squares regression; XGBoost, extreme gradient boosting; Ridge, ridge regression; LASSO, least absolute shrinkage and selection operator; KNN, k-nearest neighbors; RF, random forest; MAE, mean absolute error; RMSE, root mean squared error; R^2^, coefficient of determination.
